# General practice variation in spirometry testing among patients receiving first-time prescriptions for medication targeting obstructive lung disease in Denmark: a population-based observational study

**DOI:** 10.1186/1471-2296-14-113

**Published:** 2013-08-07

**Authors:** Mette M Koefoed, Jens Søndergaard, René dePont Christensen, Dorte E Jarbøl

**Affiliations:** 1 Research Unit of General Practice, Institute of Public Health, University of Southern Denmark, J.B. Winsløws Vej 9A, 1, DK-5000, Odense C, Denmark

**Keywords:** Obstructive lung disease, Spirometry, Practice characteristics

## Abstract

**Background:**

Spirometry testing is essential to confirm an obstructive lung disease, but studies have reported that a large proportion of patients diagnosed with COPD or asthma have no history of spirometry testing. Also, it has been shown that many patients are prescribed medication for obstructive lung disease without a relevant diagnosis or spirometry test registered. General practice characteristics have been reported to influence diagnosis and management of several chronic diseases. However, these findings are inconsistent, and it is uncertain whether practice characteristics influence spirometry testing among patients receiving medication for obstructive lung disease. The aim of this study was therefore to examine if practice characteristics are associated with spirometry testing among patients receiving first-time prescriptions for medication targeting obstructive lung disease.

**Methods:**

A national register-based cohort study was performed. All patients over 18 years receiving first-time prescriptions for medication targeting obstructive lung disease in 2008 were identified and detailed patient-specific data on sociodemographic status and spirometry tests were extracted. Information on practice characteristics like number of doctors, number of patients per doctor, training practice status, as well as age and gender of the general practitioners was linked to each medication user.

**Results:**

Partnership practices had a higher odds ratio (OR) of performing spirometry compared with single-handed practices (OR 1.24, CI 1.09-1.40). We found a significant association between increasing general practitioner age and decreasing spirometry testing. This tendency was most pronounced among partnership practices, where doctors over 65 years had the lowest odds of spirometry testing (OR 0.25, CI 0.10-0.61). Training practice status was significantly associated with spirometry testing among single-handed practices (OR 1.40, CI 1.10-1.79).

**Conclusion:**

Some of the variation in spirometry testing among patients receiving first-time prescriptions for medication targeting obstructive lung disease was associated with practice characteristics. This variation in performance may indicate a potential for quality improvement.

## Background

Spirometry is recommended for diagnosis and management of obstructive lung diseases like asthma and chronic obstructive pulmonary disease (COPD) [1-3]. Spirometry testing is not only essential to confirm a diagnosis of obstructive lung disease, it also enables the general practitioner (GP) to rule out airway obstruction in patients with respiratory symptoms caused by other illnesses, such as heart failure or lung cancer.

Despite international guidelines recommendations, we confirmed that a large proportion of patients prescribed medication targeting obstructive lung diseases do not undergo spirometry testing [4]. Hence, these patients may be medicated without having airway obstruction and exposed to unnecessary economic costs and medication risks [5,6]. More important, when spirometry is not performed, patients may experience an unnecessary delay in the diagnostic process. In Denmark, the majority of patients with respiratory symptoms are diagnosed and managed in general practice. Spirometry has been shown to be both feasible and reliable in general practice [7], but if preferred, GPs can also refer patients to spirometry testing at hospitals or outpatient clinics. Underutilisation of spirometry when diagnosing obstructive lung disease is well known [8-11]. Patient characteristics like age and gender have been shown to influence spirometry testing [4,11,12] and accuracy of diagnosis [13]. Also, some doctor and practice characteristics have been shown to influence spirometry testing; unfamiliarity with conducting or interpreting spirometry tests and spirometry being too time-consuming are reported as barriers [14-17], and practice characteristics like presence of a practice nurse and use of protocols have been reported to enhance spirometry testing [15]. Rural differences in spirometry testing have also been reported [18].

Studies have reported practice characteristics such as practice size, organisation in partnership or single-handed practices and having training practice status to influence diagnosis and management of other illnesses [19-21]. Doctor characteristics like age and gender have also been associated with different practice patterns [22,23]. However, we have not found studies assessing these factors association with spirometry testing. Identifying practice characteristics may have important implications for future organisation of primary care services [24] and can help target interventions aiming to improve spirometry testing. The aim of this study was therefore to examine if variation in spirometry testing among patients receiving first-time prescriptions for medication targeting obstructive lung disease is associated with specific practice characteristics.

## Methods

A register-based cohort study covering the entire population of 5.5 mill people and all general practices in Denmark (approx. 2400) was carried out. More than 98% of the population in Denmark is registered with a general practitioner, who provides primary care services, acts as a gatekeeper and refers patients to specialist care when needed. The health care system in Denmark is tax funded and patients have free access to all services related to general practice and hospital care, including spirometry [25]. All general practices have direct access to spirometry testing; either in their practice where the doctors can conduct these tests themselves or have practice staff conduct spirometry testing or the doctors can refer patients to spirometry testing at hospitals or outpatient clinics. From an earlier study we know that the majority of spirometry tests conducted among new medication users were performed in general practice [4].

All Danish citizens are registered in the Danish Civil Registration System and assigned a unique personal identification number. Likewise, each general practice is also assigned a unique identification number and these identification numbers are used in all national registers, enabling accurate linkage between patients, health care services and general practice [26].

This study links several national registers all maintained in Statistics Denmark, where researchers can apply for access.

### Study subjects

Patients were identified in the National Prescription Register. We identified all adults who were first-time users of medication targeting obstructive lung disease in 2008. Firstly, all patients who redeemed medication targeting obstructive lung disease, defined as the anatomical therapeutic chemical (ATC) code R03 in 2008, were identified. We then excluded patients who were either under 18 years of age on 1 January 2008 or who had previous records of prescriptions with ATC code R03 in the register (1995–2007). All medication with ATC code R03 requires a prescription and registration is therefore complete. For each patient we identified whether they had redeemed R03 medication repeatedly within the first year and how many types of R03 medication they initiated within this first year. These two variables, “redeemed repeatedly” and “number of therapies”, were used as proxies for severity. Additionally, for each patient we retrieved 2008 data on socioeconomic and demographic status such as age, gender, income, highest attained education, labour market affiliation and cohabitation status.

### Outcome - spirometry within the first year when initiating medication

All spirometry measurements registered in the time period 2007–2009 were extracted from the National Health Service Register, which covers primary care, and from the National Patient Register, which covers hospitals and outpatient clinics. These registers are administrative databases used for reimbursement and a prerequisite for reimbursement is that all services conducted, including spirometry testing, must be recorded in these registers. For each patient we assessed if spirometry was registered in an 18-month period counting from 6 months before to 12 months after the date of the first redemption of obstructive lung medication. All spirometric procedures were included, irrespective of whether they were performed in general practice, in an outpatient clinic, or in a hospital. The results from the spirometry tests were not available in the register.

### General practice

All data on general practice were extracted from the Danish National Health Service Provider Register. We extracted data covering the period July 2007 – December 2009, corresponding to the absolute observation time of the cohort. A total of 428 practices were omitted due to missing data at the beginning or end of the time period, indicating that these practices were established or closed in this time period. A further 11 practices were omitted due to a small list size (<500 patients), because these practices are probably atypical and are not representative of general practice. For each general practice we identified the number of established doctors registered at each practice. Doctors not registered in the entire period were defined temporary doctors and were not considered to be in the established doctor group. Practices were defined single-handed practices if only one established doctor was registered, and partnership practices if two or more established doctors were registered. The majority of the temporary doctors in general practice were junior doctors having six months’ residency in practice, and practices with these doctors listed in the time period were defined training practices. The number of patients per doctor was defined as the practice’s patient list size divided by the number of established doctors. In single-handed practices the doctor’s age and gender were extracted, in partnership practices we calculated the mean age of the established doctor group and assessed whether their gender was exclusively male or female, predominantly male or female or equally mixed. For each practice we calculated a “spirometry proportion” defined as the proportion of adult patients within the practice receiving first-time prescriptions for medication targeting obstructive lung disease who had spirometry performed in the 18-month interval.

### Statistical analysis

Practice characteristics are reported as categorical variables. For each practice characteristic we report the mean and standard deviation of the “spirometry proportion”.

We used mixed effects logistic regression models with patients nested within practice to calculate odds ratios (ORs) with 95% confidence intervals (CI) for the associations between practice characteristics and having spirometry performed. We used two models. Model one estimated the crude OR for each practice characteristic’s association with spirometry testing, model two estimated the OR for each practice characteristic, adjusted for both patient characteristics and the other practice characteristics included in the analysis. Our primary analysis was model 2. Analyses comprised the entire cohort of general practices and were subsequently stratified into single-handed and partnership practices. This stratification was done for two reasons: firstly, we hypothesised that this important organisational factor could interact with other practice characteristics, and secondly, some of the variables like age and gender were average values in partnership practices, but precise values in single-handed practices, and separate analyses were needed. Patient characteristics adjusted for were age, gender, income, highest attained education, labour market affiliation, cohabitation status, number of therapies initiated in the first years and repeat prescription redemption. P-values < 0.05 were considered statistically significant associations. Finally, we conducted subgroup analyses of the association between practice characteristics and spirometry testing among two different subgroups of patients 1) patients over 45 years of age initiating at least two types of medication and redeeming medication repeatedly and 2) patients less than 45 years of age initiating only one type of medication. This was done to assess if the associations shown among practice characteristics in the overall group of patients receiving first-time prescription for medication targeting obstructive lung disease were also present in 1) a subgroup of patients where COPD is more common and 2) among younger patients with mono therapy. We also repeated all analyses including peak flow measurements conducted in the time period as peak flow measurements might have been used in asthma patients and this might influence some of associations seen.

All statistical analyses were carried out using STATA 11 (STATACorp, College Station, TX, USA).

### Ethics

This project is register-based and according to “The Act on Research Ethics Review of Health Research Projects in Denmark” only questionnaire surveys and medical database research projects involving human biological material are required to be notified to the research ethics committee. The research ethics committee has, therefore, not been contacted. The study was approved by the Danish Data Protection Agency, J.nr. 2011-41-5798.

## Results

**Figure 1:**
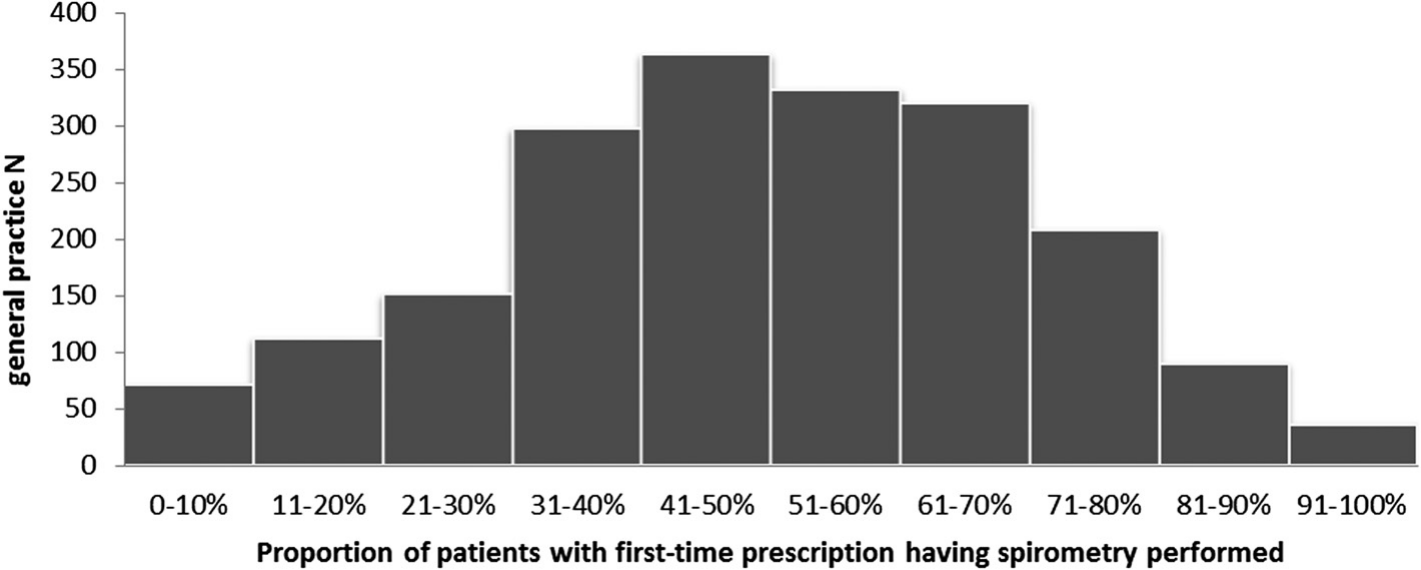
Distribution of the spirometry proportion among general practice in total numbers (N=1980).

A total of 1980 practices and 35 677 patients were included in our analysis. Just about half of the patients had spirometry performed in the time period corresponding to 51.2% (18 263/35 677). Among general practices, the mean “spirometry proportion” was 50.8%. The distribution of the “spirometry proportion” among general practice is illustrated in Figure 1 and it demonstrates quite a large variation between practices. An overview of practice characteristics and their mean “spirometry proportion” is shown in Table 1.

**Table 1:** Distribution of practice characteristics within the entire general practice cohort in absolute numbers (N); the mean and standard deviation of the variable “spirometry proportion”* is reported for each practice characteristic

		**All general practices**		**Single-handed practices**		**Partnership practices**	
		**N**	**Mean (SD)**	**N**	**Mean (SD)**	**N**	**Mean (SD)**
Partnership practice	Yes	773	54.4 (16.8)	-	-	773	54.4 (16.8)
	No	1207	48.6 (22.7)	1207	48.6 (22.7)	-	-
Training practice	Yes	566	53.7 (18.0)	239	53.8 (20.2)	327	53.7 (16.1)
	No	1414	49.7 (21.8)	968	47.3 (23.2)	446	54.8 (17.3)
No of doctors	1	1207	48.6 (22.8)	1207	48.6 (22.8)	-	-
	2	388	54.2 (18.7)	-	-	388	54.2 (18.7)
	3	213	53.4 (15.6)	-	-	213	53.4 (15.6)
	4	94	57.2 (13.2)	-	-	94	57.2 (13.2)
	5	52	54.5 (14.2)	-	-	52	54.5 (14.2)
	>5	23	55.0 (11.3)	-	-	23	55.0 (11.3)
Age							
(mean for partnership practices)	<45	106	56.0 (19.1)	67	52.2 (18.8)	39	62.5 (18.1)
	45–49	238	55.8 (18.1)	122	54.5 (20.0)	116	57.2 (15,8)
	50–54	516	54.2 (18.8)	228	52.4 (21.4)	288	55.7 (16.3)
	55–59	609	49.7 (20.9)	366	48.3 (23.3)	243	51.7 (16.4)
	60–64	390	46.4 (22.4)	314	45.9 (23.3)	76	50.4 (17.8)
	>65	121	41.2 (23.9)	110	40.7 (24.3)	11	46.6 (−)
Gender	Male	1017	49.4 (22.1)	873	48.7 (22.7)	144	53.4 (17.5)
Predominantly male		189	54.4 (15.0)	-		189	54.4 (15.0)
Equal male/female		283	54.9 (18.6)	-		283	54.9 (18.6)
Predominantly female		98	54.3 (13.6)	-		98	54.3 (13.6)
	Female	393	49.2 (22.3)	334	48.3 (23.0)	59	54.0 (17.1)
Patients per doctor	<1347	513	49.8 (22.8)	227	43.9 (21.8)	286	54.4 (18.2)
	1347–1575	489	51.0 (19.8)	277	49.1 (21.8)	212	53.5 (16.4)
	1576–1756	489	52.3 (20.8)	307	49.9 (23.4)	182	56.5 (14.6)
	>1756	489	50.3 (19.7)	396	49.9 (20.3)	93	51.9 (17.0)

*The “spirometry proportion” is defined as the proportion of adult patients within the practice receiving first-time prescriptions for medication targeting obstructive lung disease who had spirometry performed in the 18-month interval.

When comparing all general practices, partnership practices had a higher OR of performing spirometry compared with single-handed practices (OR 1.24, CI 1.09-1.40), Table 2. In all analyses we saw that increasing age among the group of established doctors decreased the odds of spirometry testing; in the analysis comparing all practices, the smallest OR was seen among doctors over 65 years (OR 0.33, CI 0.22-0.50). The most pronounced effect of doctors’ increasing age on spirometry was seen among partnership practices (OR 0.25, CI 0.10-0.61), Table 3. A test for trend showed a significant association between increasing GP age and decreasing spirometry testing. Being a training practice was significantly associated with spirometry testing among single-handed practices (OR 1.40, CI 1.10-1.79), Table 4. There was no significant association between the doctors’ gender or number of patients per doctor and having spirometry performed. Further, there was no significant association between number of doctors in a partnership practice and having spirometry performed. Both subgroup analyses demonstrated the same tendency in associations: an increased OR for spirometry testing was seen among partnership practices, practices with younger doctors and among single-handed practices with training practice status (data not shown). These associations were however, only statistically significant among patients over 45 years of age initiating at least two types of medication and redeeming medication repeatedly. Adding peak flow measurements to the analyses did not influence the associations significantly.

**Table 2:** Association between practice characteristics and spirometry testing among all practices

		**Model 1**	Model 2**
		**Crude OR**	**Adjusted OR**
		**(95% CI)**	**(95% CI)**
Training practice			
	No	1	1
	Yes	1.20 (1.06–1.36)*	1.10 (0.97–1.25)
Single-handed practice			
	Yes	1	1
	No	1.34 (1.16–1.55)*	1.24 (1.09–1.40)*
Mean age of doctors (years)			
	≤ 45	1	1
	45–49	0.94 (0.74–1.19)	0.87 (0.66–1.14)
	50–54	0.88 (0.70–1.09)	0.78 (0.60–1.00)
	55–59	0.68 (0.53–0.87)*	0.58 (0.44–0.76)*
	60–64	0.58 (0.43–0.79)*	0.52 (0.39–0.70)*
	≥65	0.41 (0.27–0.64)*	0.33 (0.22–0.50)*

*P-value< 0.05 **Adjusted for patient factors and practice characteristics.

**Table 3:** Association between practice characteristics and spirometry testing in partnership practices

		**Model 1**	Model 2**
		**Crude OR**	**Adjusted OR**
		**(95% CI)**	**(95% CI)**
Training practice			
	No	1	1
	Yes	0.95 (0.84–1.08)	0.91 (0.79–1.04)
Mean age of doctors (years)			
	≤ 45	1	1
	45–49	0.72 (0.50–1.03)	0.66 (0.45–0.97)*
	50–54	0.68 (0.47–0.98)*	0.61 (0.42–0.89)*
	55–59	0.54 (0.34–0.86)*	0.45 (0.29–0.71)*
	60–64	0.52 (0.31–0.86)*	0.43 (0.26–0.72)*
	≥65	0.39 (0.17–0.90)*	0.25 (0.10–0.61)*
Number of doctors			
	2	1	1
	3	0.97 (0.84–1.13)	0.99 (0.77–1.27)
	4	1.17 (0.95–1.45)	1.15 (0.90–1.45)
	5	1.03 (0.82–1.30)	1.08 (0.77–1.51)
	>5	1.05 (0.76–1.37)	1.03 (0.69–1.53)
Number of patients per doctor			
	<1347	1	1
	1347–1575	0.96 (0.82–1.12)	0.97 (0.82–1.15)
	1576–1756	1.12 (0.94–1.34)	1.16 (0.96–1.34)
	>1756	0.86 (0.69–1.07)	0.88 (0.70–1.11)
Gender of doctors			
	Male	1	1
	Predominantly male	1.05 (0.87–1.27)	0.99 (0.77–1.27)
	Equal male/female	1.07 (0.89–1.29)	1.04 (0.85–1.28)
	Predominantly female	1.05 (0.84–1.32)	0.94 (0.73–1.26)
	Female	1.07 (0.81–1.42)	1.04 (0.76–1.42)

*P-value< 0.05 **Adjusted for patient factors and practice characteristics.

**Table 4:** Association between practice characteristics and spirometry testing in single-handed practices

		**Model 1**	Model 2**
		**Crude OR**	**Adjusted OR**
		**(95% CI)**	**(95% CI)**
Training practice			
	No	1	1
	Yes	1.40 (1.06–1.87)*	1.40 (1.10–1.79)*
Age of doctor (years)			
	≤ 45	1	1
	45–49	1.11 (0.78–1.58)	1.09 (0.73–1.61)
	50–54	0.99 (0.78–1.58)	0.96 (0.67–1.38)
	55–59	0.79 (0.73–1.35)	0.71 (0.49–1.03)
	60–64	0.69 (0.56–1.10)	0.64 (0.43–0.95)*
	≥65	0.50 (0.28–0.89)*	0.44 (0.28–0.76)*
Number of patients			
	<1347	1	1
	1347–1575	1.29 (0.97–1.71)	1.26 (0.95–1.67)
	1576–1756	1.30 (0.99–1.72)	1.21 (0.92–1.59)
	>1756	1.35 (1.02–1.79)*	1.17 (0.90–1.51)
Gender of doctor			
	Male	1	1
	Female	0.98 (0.84–1.15)	0.93 (0.77–1.12)

*P-value< 0.05 **Adjusted for patient factors and practice characteristics.

## Discussion

### Main findings

This study demonstrated that patients receiving first-time prescriptions for medication targeting obstructive lung disease had higher odds of having spirometry performed if their general practice was a partnership practice. All analysis confirmed decreasing spirometry testing with increasing age of doctors. Among single-handed practices, training practice status was associated with increased spirometry testing. These associations all had an OR above 1.23 or below 0.67 and were considered relevant associations.

### Strengths and limitations of this study

The register-based design has the major strength that it allows us to include the entire population and all established general practices in Denmark. The validity of the data in these national registries is considered high, as they are based on administrative data used for reimbursement in the health care system [27]. Due to this economic incentive, spirometry recording is quite complete, although a slight under- or over-recording cannot be entirely excluded. The low rate of spirometry testing is therefore mainly due to non-use and not to inconsistent recording. The registers do, however, not contain data on how the spirometry was conducted, and we cannot exclude some variation in the quality of these measurements.

The registries enable accurate linkage of detailed information on each practice and patient and make it possible to adjust for numerous patient factors, enhancing the possibility of isolating and assessing practice characteristics’ influence on spirometry testing in our cohort. Nonetheless, it is important to remember that influence of patient characteristics cannot be entirely excluded; the registers cannot provide complete information on all sociodemographic patient characteristics.

Another challenge was that patient data could only be linked on the level of general practice, preventing us from identifying the doctor within the practice who is primarily responsible for each patient. This complicates the assessment of the influence of doctors’ age and gender on spirometry testing when dealing with partnership practices. Mean age of established doctors is a compromise and is not as informative as an individual doctor’s age. Also, “patients per doctor”, a proxy for workload, may be inaccurate, as doctors in Denmark can schedule their own work. General practitioners with few listed patients may work part-time and still have a high workload in practice.

Newly established and closing practices were excluded in these analyses, and it is important to remember that our data underrepresent these practices, but this was done deliberately. Firstly, forming and closing practices were quite unstable in the time period with regard to both number of doctors and number of patients, making categorisation quite difficult, and secondly, we hypothesised that forming and closing practices could confound our results in favour of larger practices.

Other potential influential variables could have been interesting to include in our study if they were available in our databases. The presence of a practice nurse and the practice’s location (rural or urban area, distance to outpatient clinics) could influence spirometry testing and were very relevant to include in our study. However, the registers contain no data on employed staff in general practice, and the limited data on practice location were not adequate for assessing either rural or urban location or distance to relevant outpatient clinics.

### Interpretation of findings in relation to previously published work

Two studies tested if quality of care scores in asthma patients were influenced by practice size, but found no association [28,29]. Other studies have found single-handed practices and small practice size to be associated with increased acute admission rates to hospitals for asthma, but not for COPD [30,31]. Our measure for practice size was divided into two variables: number of doctors and number of patients per doctor. When looking solely at the number of doctors, we found that single-handed practices had lower odds of performing spirometry compared to partnership practices in concordance with the above mentioned studies. Among partnership practices, however, there was no association between number of doctors and odds of spirometry testing, indicating that size of partnership practices was not associated with spirometry testing. Further, we found no association between number of patients per doctor and spirometry testing. Although partnership practices and larger practices have been associated with higher scores for quality of care in several chronic diseases [19,20], studies are not consistent with regard to this issue, as the opposite has also been shown [32], and it is interesting that patient satisfaction has been reported to be in favour of single-handed practices [33,34].

Increasing age among doctors has been reported to be associated with decreasing quality of care scores in studies [35,36] and these findings are in concordance with our study, where we found a clear tendency between increasing age and decreasing OR for spirometry testing. Our study does not clarify why older doctors perform fewer spirometry tests in patients initiating medication, but general practitioners’ age has been shown to influence clinical practice patterns, with older GPs providing more home visits, doing fewer procedures and having higher prescribing rates [22]. We found no association between GP gender and spirometry testing. Other studies have reported that when assessing quality scores, female physicians are more often among high scorers and the majority of the lowest scoring physicians are men [35,37]. Specifically, female GPs have been reported to attain higher scores in evaluation of antenatal care and more often refer to bone mineral density testing [23,38]. We therefore hypothesised that female GPs performed more tests as shown by Ioannidis et al. [23], but our data showed no indication of this pattern.

Training practices have also been shown to influence quality of care [19,35] and in our study we also saw this tendency, but only among single-handed practices. Why training practice status influences single-handed practices, but not partnership practices, is unknown, but we suggest that this difference in effect is due to a greater interaction between the single-handed practitioner and the resident doctor compared to the interaction seen in a partnership practice with several doctors.

Overall, we conclude that the variation in spirometry testing between practices was quite large and some of this variation can be associated with practice characteristics. Concluding whether the variation shown in spirometry testing is due to a variation in quality of care is more challenging. Although spirometry is essential for diagnosing obstructive lung disease and could therefore be used as a marker of good quality, it may not be relevant for all patients receiving first-time prescriptions for medication targeting obstructive lung disease to have spirometry performed. Among some patients it may be clinically meaningful not to conduct spirometry testing, for example among patients who are unable to corporate sufficiently. However, the variations shown could indicate a potential room for quality improvement and further studies should be conducted to clarify this issue. Also, assessing changes in spirometry testing over time in general practice would be relevant, as improvements have been seen in outpatient clinics in recent years [39].

## Conclusions

Some of the variations in the frequency of spirometry testing are associated with practice characteristics. Young age among doctors, being a partnership practice, or if a single-handed practice, being a training practice, were all factors associated with increased odds of performing spirometry when patients receive first-time prescriptions for medication targeting obstructive lung disease.

## Competing interests

The authors declare that they have no conflicts of interest in relation to this article.

## Authors’ contributions

MMK, RC, JS and DJ all participated in the design of the study. MMK and RC performed the statistical analysis. MMK drafted the first manuscript and all authors helped extensively revising the manuscript. All authors read and approved the final manuscript.

## Pre-publication history

The pre-publication history for this paper can be accessed here:

http://www.biomedcentral.com/1471-2296/14/113/prepub
